# Design and methodology of a cluster-randomised controlled trial on cash plus interventions for preventing child wasting in Somalia

**DOI:** 10.7189/jogh.16.04018

**Published:** 2026-02-27

**Authors:** Shelley Walton, Kemish Kenneth Alier, Sydney Garretson, Samantha Grounds, Qundeel Khattak, Marina Tripaldi, Fabrizio Loddo, Said Aden Mohamoud, Mohamud Ali Nur, Sadiq Mohamed Abdiqadir, Emily Mitchell, Mohamed Billow Mahat, Maimun Gure, Dahir Isaq Jibril, Dahir Gedi, Michael Ocircan, Meftuh Omer Ismail, Abdullahi Abdulle Farah, Abdifatah Ahmed Mohamed, Adam Abdulkadir Mohamed, Nadia Akseer

**Affiliations:** 1 Johns Hopkins University, Department of International Health, Baltimore, Maryland, USA; 2 Save the Children, Save the Children International, London, UK; 3 Save the Children, Somalia Country Office, Mogadishu, Somalia; 4 Federal Government of Somalia, Ministry of Health & Human Service, Mogadishu, Somalia

## Abstract

**Background:**

Somalia is a conflict, flood, and drought prone country with high rates of food insecurity and child wasting. Save the Children partnered with Johns Hopkins University to study the most effective and cost-effective combinations of assistance to prevent acute malnutrition among pregnant and lactating women (PLW) and children under five (CU5) in a six-month humanitarian programme. This study implemented an cluster-randomised controlled trial (RCT) using adaptive design methodology to: (1) estimate and compare wasting incidence and prevalence of CU5 and their mothers receiving on a monthly basis either cash (Arm 1), cash + social and behaviour change communication (Arm 2), or cash + top-up cash (Arm 3); after three months and six months; (2) calculate the costs and cost-effectiveness of the different intervention arms; (3) understand perspectives and experiences of mothers and fathers of CU5 beneficiaries; (4) monitor the functionality of markets and availability and prices of foods. This paper presents the approach that was designed and implemented to study these objectives.

**Methods:**

This study employed a mixed-methods approach with a quantitative component conducted at three time points to collect anthropometric measurements and household survey data. Primary outcomes, such as child and maternal wasting, were assessed using standardised World Health Organization criteria. Additional data on food security, maternal and child health, and household conditions were collected to evaluate immediate, underlying, and basic causes of malnutrition. Cost analyses evaluated programmatic and societal costs of the intervention. An adaptive trial design was implemented, allowing the methodology to evolve as new challenges emerged.

**Conclusions:**

This trial applied an adaptive mixed methods design to evaluate the effectiveness and cost-effectiveness of cash assistance interventions in Somalia, overcoming complex humanitarian operational challenges. Strong partnerships and flexible trial design allowed us to adjust to unpredictable events and maintain research rigor. These findings highlight the value of adaptive designs and mixed methods for improving child nutrition outcomes in complex settings.

**Registration:**

The cluster-RCT is registered at ClinicalTrials.gov, ID: NCT06642012.

In Somalia, acute malnutrition case admissions among children under five (CU5) have risen dramatically in recent years [1]. Due to climate shocks (prolonged drought and flooding), persistent conflict, and global supply and price shocks, millions of people in Somalia are suffering from food insecurity and experiencing one of the most complex humanitarian crises [2]. The Food Security and Nutrition Analysis Unit’s (FSNAU’s) recent survey showed that at least 1.5 million children under five (42.8% of the total under-five children in Somalia) and 4.3 million people overall are projected to be acutely malnourished from August 2023 through July 2024, with a projected 330 630 children being severely malnourished [3].

In response to this crisis, humanitarian actors have implemented interventions such as in-kind food aid, specialised nutritious foods, and increasingly, cash and voucher assistance (CVA). CVA has become a preferred modality in many settings due to its potential flexibility, cost-efficiency, and support for local markets. However, there remains limited evidence on how CVA can be most effectively designed and targeted to improve nutrition outcomes in fragile contexts like Somalia. Specifically, questions remain about the optimal amount, duration, and combinations of CVA with other interventions such as social and behaviour change communication (SBCC) or additional top-up cash [4–7].

Evidence gaps identified by Elrha evidence reviews in 2015 [4] and 2022 [5], a research agenda by the World Health Organization (WHO) in 2018 [6], and research questions emphasised by the United Nations International Children’s Emergency Fund (UNICEF) in 2021 [7] highlight the need for further research to explore:

1) CVA duration and amount,

2) cost-effectiveness design, and

3) evidence generation in humanitarian settings.

These gaps underscore the critical need for targeted research to inform policy and practice, ensuring that humanitarian interventions are both effective and cost-effective in addressing malnutrition in crisis-affected populations.

The unpredictable context of humanitarian settings due to climate or conflict-related events can make it challenging for researchers to study these questions. There is an evidence gap in study methodologies and approaches, especially for mixed-methods impact evaluations and cost analyses, that can provide reliable data to answer these questions under the variable conditions often present in crisis settings [8–12]. Adaptive design methods in humanitarian settings provide a flexible approach where interventions or programmes are modified based on real-time data and feedback gathered from the study setting and community throughout the project [13–15]. Adaptive designs allow research and implementers to better respond to changing needs and circumstances in dynamic humanitarian crises. Methodologies that adapt to complex research environments, highlight lessons learned and best practices for researchers, and fill evidence gaps are critical to publish to support future research in humanitarian settings.

To address these evidence gaps, we conducted a prospective, cluster-randomised controlled trial in two high-malnutrition regions of Somalia, Bay, and Hiran [1,16], to evaluate three cash-based intervention strategies. These strategies were co-developed with implementing partners and local stakeholders, based on prior programming experience and guidelines [17]. This trial followed CU5 and pregnant and lactating women (PLW) to assess the effectiveness and cost-effectiveness of combinations and durations of monthly cash assistance in preventing severe acute malnutrition (SAM) and moderate acute malnutrition (MAM) in a six-month humanitarian programme [1,16]. This three-arm trial with randomisation at the village level used a mixed methods approach, including both quantitative and qualitative data collection and analyses and cost-effectiveness analyses.

Specifically, the primary aims of this trial were to:

1) to estimate and compare wasting incidence and prevalence of children 6–59 months-old and their mothers receiving on a monthly basis either cash (Arm 1 – control), cash + social and behaviour change communication (SBCC) (Arm 2), or cash + top-up cash (Arm 3); after three months and six months of cash assistance;

2) to calculate the costs and cost-effectiveness of the different intervention arms;

3) to understand perspectives and experiences of mothers and fathers of CU5 beneficiaries of the cash programme;

4) to monitor the functionality of markets and availability and prices of foods.

The purpose of this paper is to describe the adaptive trial design and mixed-methods approach used to evaluate the effectiveness, cost-effectiveness, and feasibility of different cash assistance strategies for preventing acute malnutrition in Somalia; outcome results are presented in a separate publication.

The methodology was designed to address the evidence gap and inform global guidance on the duration of cash for nutrition programmes, the amount of cash to prevent acute malnutrition, and the optimal combinations of interventions in cash for nutrition programmes. The published research methodology, study instruments, and lessons learned offer a novel example of conducting rigorous research in a complex humanitarian setting, and aim to support future studies and cash-for-nutrition programme design in Somalia and similar crisis-affected contexts by sharing practical, evidence-based strategies.

## METHODS

### Trial setting

The two trial sites were Baidoa and Beledweyne, the capitals of the Bay and Hiran regions, respectively, and were primarily rural [18,19]. Bay and Hiran were selected as trial sites not only due to existing Save the Children (SC) and Bureau for Humanitarian Assistance (BHA) Cash Plus for Nutrition programming, but also due to their high global acute malnutrition (GAM) and severe acute malnutrition (SAM) rates, with GAM rates of 18.3% and SAM rates of 3.6% in Beledweyne district, and GAM rates of 19.1% and SAM rates of 4.9% in Baidoa-Buurhakaba livelihood zones [3]. Bay and Hiran are located in the southwest region of Somalia. In 2021, the population of Bay was around 1.1 million people, while Hiran had an estimated 427 124 people [20,21].

Both Bay and Hiran have been greatly affected by climate crises (droughts, floods), instability, and conflict that have affected livelihoods and contributed to the displacement of thousands [22]. Over the years, such insecurities have contributed to the displacement of over 25 000 people from Bay and over 250 000 people from Hiran from July 2021 to November 2022 [23–26]. In 2023, Hiran received an influx of nearly half a million new internally displaced persons (IDP), and Bay received 219 000 [26]. Roughly 25% of the country’s IDP sites are around the Baidoa area in Bay, and these sites experience some of the highest levels of food insecurity in Somalia [27]. According to the Integrated Food Security Phase Classification, in the first quarter of 2023, roughly 750 000 individuals in Bay suffered from level 3 (crisis) or level 4 (emergency) food insecurity [1].

### Conceptual framework

The UNICEF Conceptual Framework on Maternal and Child Nutrition categorises the determinants of malnutrition into three groups: immediate, underlying, and basic/enabling causes [28,29]. This evidence-based conceptual framework informed the trial design and both quantitative and qualitative data collection and analysis (Figure S1 in the **Online Supplementary Document**).

### Study design

This adaptive trial used a mixed methods design inclusive of a difference-in-difference approach for advanced quantitative analysis, qualitative data collection (via focus group discussions), market monitoring surveys and detailed cost, cost efficiency and cost-effectiveness analysis. The research team utilised an explanatory sequential design to integrate the qualitative study components, collecting and analysing quantitative data first, followed by qualitative data. We then used the qualitative data to help explain and contextualise the quantitative findings.

### Trial methodology development and adaptive design

This trial methodology was co-developed by researchers at Johns Hopkins University (JHU), programme staff at Save the Children International and Save the Children Somalia, and other global experts on nutrition assistance in humanitarian settings through iterative consultations. The methods described in this paper are the final version of the trial design.

**Figure 1:**
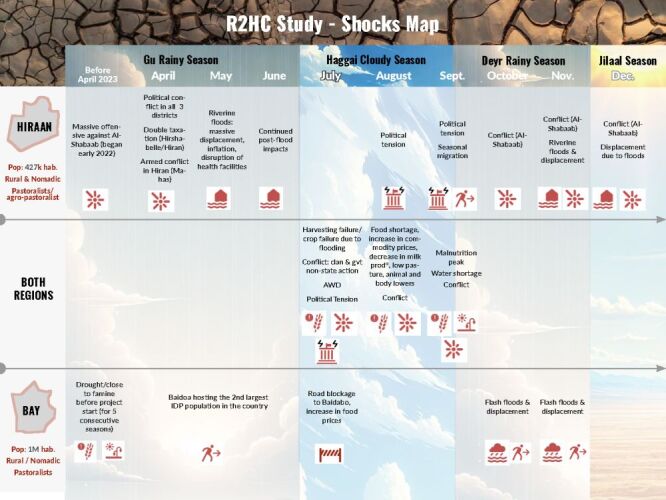
Bay and Hiran 2023 shocks map.

An adaptive design for the trial was adopted to account for the variable humanitarian setting and to ensure the validity and integrity of data collection [30]. For example, severe flooding occurred throughout Somalia and in Bay and Hiran during the trial, affecting almost 2.5 million people and displacing over one million individuals [25]. The flooding resulted in displacement and disrupted access to research sites, affecting data collection. However, the trial team was able to adapt and respond to the crisis in a way that maintained the integrity of the research. The timeline of conflict and climate-related shocks to the study setting is found in **Figure 1**.

### Trial design

The prospective cluster-randomised controlled trial (cluster-RCT) was designed to study combinations of cash assistance across three study arms. A cluster-RCT design was selected because the interventions were implemented at the community level, making individual randomisation impractical and increasing the risk of contamination between participants within the same geographic area. The base cash assistance amounts were established by the National Cash Working Group based on the Minimum Expenditure Basket (MEB). Cash amounts were calculated to meet 80% of a person’s energy needs (kcals) per month and were based on the average number of household members; six to seven members in the Somalia context [31]. Amounts distributed considered fluctuations in food prices due to conflict, climate shocks, and inflation. They allowed for basic food purchases like staple grains, legumes, oil, and vegetables, and occasional purchases of fresh fruits, dairy products, and meat products. The unconditional cash (CVA) for food assistance was designed to increase purchasing power, thereby meeting the immediate needs of most food-insecure people in the target regions. This integrated approach also aimed to prevent child malnutrition and wasting relapse.

The combinations of assistance studied in three separate arms were:

– Arm 1: CVA to targeted families – one mobile transfer per month for six consecutive months. Households in Bay received 90 USD per month and households in Hiran received 70 USD per month;

– Arm 2: CVA with SBCC – SBCC package included interpersonal communication, such as one-to-one consultations for mothers of CU5, bi-monthly group sessions of approximately 45–60 minutes through the Mother-to-Mother Support Groups on key topics related to nutrition, health, and community awareness raising campaigns (Text S2 and Table S14 in the **Online Supplementary Document**). Households in Bay received 90 USD per month and households in Hiran received 70 USD per month;

– Arm 3: CVA with additional monthly nutrition-cash top up – 1 per month for 6 months. Households in Bay received 90 USD plus an additional 35 USD top-up (125 USD total) per month and households in Hiran received 70 USD plus an additional 35 USD top-up (105 USD total) per month.

The programme was implemented from June to November 2023. Baseline quantitative data collection occurred in May 2023, midline occurred in September 2023, and endline occurred in December 2023. Qualitative data were collected in January 2024.

### Trial population and sampling methods

The trial participants were comprised mothers, fathers and their children (CU5) whose households were enrolled in the BHA Cash Plus for Nutrition programme. 

For CU5 and their mothers, the study inclusion and exclusion criteria are found in **Table 1**.

**Table 1:** Trial inclusion and exclusion criteria

Study participant	Inclusion criteria	Exclusion criteria
Mothers of children six to 59 mo		
	Enrolled in Save the Children Cash Plus for Nutrition program	Currently receiving treatment for MAM or SAM
	Have a child that is 6–59 mo of age at baseline	Had an episode of severe wasting (SAM) in the past 12 mo
	Subject who is voluntarily willing to participate by signing the consent form	
Children 6–59 mo		
	Enrolled in Save the Children Cash Plus for Nutrition program	Receiving treatment for MAM or SAM at baseline
	6–59 mo of age at baseline	Had an episode of severe wasting (SAM) in the past 12 mo
	Their mother is enrolled in the study	

MAM – moderate acute malnutrition, SAM – severe acute malnutrition

Prior to baseline data collection, the SC Somalia project team provided a household beneficiary register for the Cash Plus for Nutrition programme to the research team. The research team reviewed the registration details to generate a list of all CU5 mothers with their CU5s that met the inclusion and exclusion criteria. This produced the sampling frame from which the study sample was selected.

Initially 44 total clusters (villages) were assessed for eligibility. Eleven clusters were excluded because these clusters were located in heavily flood-prone areas where households easily migrate, which would have impacted the overall sample size and data collection. The sample size estimation was based on the primary outcome for the CU5, feasibility/logistics in study settings, and a 7% minimum detectable difference in post-intervention prevalence of wasting using:

1) 20% baseline wasting (figures are transient and based on the higher prevalence of the two regions);

2) intra-class correlation coefficient (ICC) from earlier studies (0.02) [32,33];

3) average cluster size (150 households);

4) number of clusters (33 total, 11 per arm);

5) 5% significance and 80% power; and

6) CU5 per household (1.3 children).

Calculations used Stata's cluster-randomised trial commands. Covariates were selected a priori based on the UNICEF conceptual framework, including child-level factors, household factors including food security, and maternal factors.

The initial sample required was 410 households per intervention group or 533 children. Accounting for 15% attrition, the final required sample size was 471 households or 613 children per arm. Thirty-three clusters were randomised to the three study arms. Each cluster ranged in size from 55 to 431 households, with an average cluster size of 140–158 households. A total of 4838 households were evaluated for eligibility against the trial’s inclusion and exclusion criteria. Of these, 3348 households were excluded because they did not have CU5 in the household or did not meet one or more exclusion criteria. Overall, 1490 total households were enrolled in the study. The final child sample was 1894 children.

**Figure 2:**
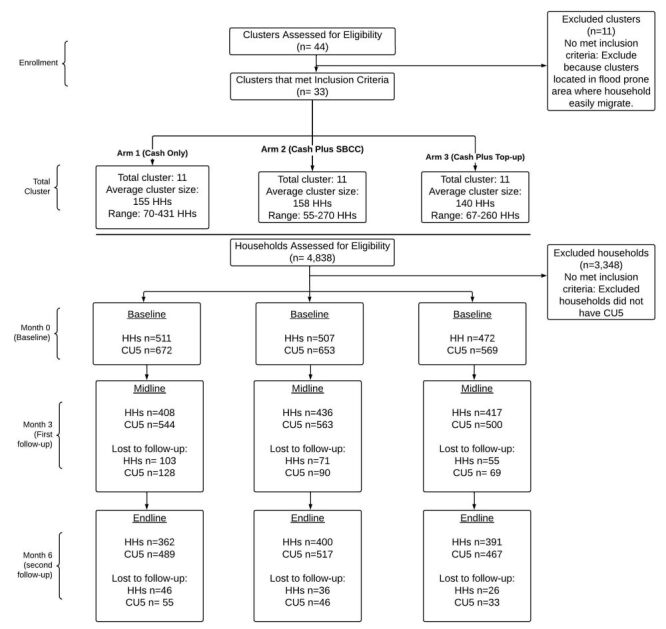
Trial recruitment consort diagram. Reasons for loss to follow-up: Household moved to another location. Household displacement due to floods. CU5 – children under five years old, HH – household.

Household and mothers of CU5 were selected from health facility registries and were then contacted by phone for study recruitment and mobilisation for data collection. **Figure 2** shows the trial recruitment and data collection sample for each study time point. Table S3 in the **Online Supplementary Document** also shows a table of subject recruitment for all study activities.

## RESULTS

### Quantitative approach

Once enrolment criteria were confirmed and informed consent was obtained, a team of trained enumerators (in pairs) conducted the household survey and collected anthropometric data for the CU5 mother and the youngest CU5 in the household. Anthropometric measures and household surveys were administered to 1490 households at baseline, 1261 households at midline, and 1153 households at endline.

#### Primary outcomes and aim of treatment comparison

The primary aim of the trial was to assess the impact of different cash assistance interventions on reducing child and maternal wasting over a six-month period. Accordingly, the primary outcomes of interest were wasting in children under five (CU5), measured using mid upper arm circumference (MUAC), weight-for-height z-scores (WHZ), and presence of oedema [34–36]. Wasting was also measured in mothers of CU5 using MUAC.

#### Secondary outcomes

A secondary outcome of interest was stunting among CU5, assessed using height-for-age z-scores (HAZ). In addition, the study explored exposures as described below.

Standard methods for anthropometric measurements were used based on the SMART methodology [37]. At each study time point, the CU5 were classified based on their outcomes according to **Table 2**.

**Table 2:** Outcome definitions for children under five (CU5) and mothers [38]

Wasting guidelines for CU5	Definitions
Without acute malnutrition	MUAC≥125 mm and WHZ≥−2, without oedema
With moderate acute malnutrition (MAM)	115 mm≤MUAC<124.9 mm and/or −3≤WHZ<−2, without oedema
With severe acute malnutrition (SAM)	MUAC<115mm, and/or WHZ<−3, and/or presence of oedema
**Stunting guidelines for CU5**	**Definitions**
Without stunting	HAZ>−2
Moderate stunting	−3<HAZ≤−2
Severe stunting	HAZ≤−3
**Malnutrition guidelines for mothers of CU5**	**Definitions**
Overweight	MUAC>300mm
Normal	MUAC≥230 mm
With moderate acute malnutrition (MAM)	MUAC<230 mm

CU5 – children under 5, HAZ – height-for-age z-score, MAM – moderate acute malnutrition, MUAC – mid-upper arm circumference, SAM – severe acute malnutrition, WHZ – weight-for-height z-score

If a CU5 or CU5 mother was identified as wasted during baseline, midline or endline, he/she was referred to the nearest health centre that offered wasting treatment; however, he/she remained in the study cohort for the duration of the study.

The primary child nutrition outcomes were assessed using MUAC, WHZ, and presence of oedema to generate categorical indicator variables that align with the 2013 WHO Child Growth Standards criteria [38]. These indicator variables were used to compare child malnutrition at each time point to measure changes in wasting over the life of the study and between study arms to examine benefits of each intervention type. Intergenerational nutrition outcomes were assessed using size at birth of the youngest child (collected at baseline). Maternal nutrition outcomes were assessed using MUAC to generate categorical indicator variables (malnourished, normal, and overweight), and the proportion of malnourished mothers were compared between study arms and at each data collection time point [39].

#### Exposures

Mothers completed household quantitative surveys at baseline, midline (three months post-baseline), and endline (six months post-baseline) to establish benchmarks and explore changes related to the intended outcomes and determinants. These questionnaires were translated to Somali and administered to all study participants (mothers of CU5). There are slight variations in each questionnaire, such as incorporating questions around displacement due to the flooding that occurred in the endline questionnaire. All questionnaire versions are included in the supplementary materials (Table S4–S6 in the **Online Supplementary Document**).

The following data were collected during the household survey at each time point and were guided by the UNICEF conceptual framework of maternal and child nutrition [28,29].

Immediate Causes: child diet was measured using global infant and young child feeding (IYCF) indicators such as exclusive breastfeeding, minimum dietary diversity (MDD), minimum meal frequency (MMF), minimum acceptable diet (MAD), and consumption of different food groups (fruits/vegetables, protein sources, *etc*.) [40]. Child health history was tracked over the life of the study; mothers were asked about the CU5′s episodes of diarrhoea, pneumonia, fever or other illnesses in the last two weeks. Maternal age, MUAC, and pregnancy status were also considered immediate causes.

Underlying causes: household food insecurity was also evaluated using the Household Hunger Scale (HHS); the Food Consumption Score (FCS, a proxy for household dietary diversity); and the reduced Coping Strategies Index (rCSI, which measures the use of negative coping strategies in the absence of resources such as food or money) [41–44]. Inadequate care and feeding practices were evaluated using maternal and child health indicators including antenatal care, birth with a skilled birthing attendant, birth via C-section, childhood vaccination, care-seeking behaviours for childhood illnesses, and growth monitoring and promotion (GMP). Additionally, SBCC indicators were evaluated to assess maternal knowledge, awareness, and practices around health and IYCF. Lastly, household environment was evaluated using indicators related to household crowding, number of CU5, access to clean water, and presence of a toilet/latrine and handwashing facilities.

Intervention: participants were surveyed on whether they were receiving assistance or support from other nutrition-sensitive or cash programmes.

Basic/enabling causes: to examine the basic/enabling causes of malnutrition, indicators such as household expenditure, household and personal assets and housing materials, maternal education and empowerment/decision-making, and gender of the head of household were assessed.

A detailed description of selected exposures and their definitions is included in Table S6 in the **Online Supplementary Document**.

#### Quantitative tools and equipment

Tools and software used for quantitative data were: Kobo-Toolbox (online tool development and management platform) and KoboCollect (mobile application) for data collection and storage; CommCare for quantitative data management; STATA v. 18.0 (StataCorp, College Station, TX, USA) and SAS v. 9.4 (SAS Institute Inc., Cary, NC, USA) for quantitative analysis.

#### Quantitative analysis

Descriptive statistics were used to summarise levels and trends in key determinants and outcomes and compared across study arms. For the main outcomes, prevalence and incidences of indicators were estimated using an intent-to-treat approach and differences were calculated between baseline, midline, and endline, and across study arms. Several sensitivity analyses were conducted to assess robustness of findings:

1) prevalence *vs*. incidence analysis compared point prevalence at each time point with cumulative incidence of new wasting episodes during follow-up;

2) per protocol analysis included only participants receiving full intervention doses, compared against the primary intent-to-treat approach;

3) missing data analysis compared complete case analysis (participants with data at all time points) *vs*. available case analysis (all available data at each time point) to evaluate impact of differential attrition across study arms.

We also estimated the effect of the interventions on primary and secondary outcomes using mixed-effects linear regression models, controlling for baseline values and imbalances between groups. Our analysis targeted marginal effects to estimate population-average intervention impacts. Primary analysis used mixed-effects difference-in-difference models: logistic regression for binary outcomes and linear regression for continuous outcomes, controlling for baseline values. Interclass Correlation Coefficient (ICC) was estimated from random intercept variance. Non-convergent models would use log-binomial regression as backup though not needed in practice. A difference-in-difference analysis design was implemented to understand statistically significant changes across time points and between study arms, with risk ratios estimated at a 0.05 error margin. All primary and secondary outcome measures were analysed and reported with 95% confidence intervals The Bonferroni correction was applied to adjust the *P* values for arms comparisons to control the family-wise error rate, and *P*-values <0.05 were considered statistically significant. Additionally, we interpreted and discussed the programmatic significance of the group effect sizes, given the context of our study.

### Qualitative approach

#### Focus group discussions – CU5 mothers and husbands

The qualitative team developed the focus group discussion (FGD) guides (Texts S8–9 in the **Online Supplementary Document**) in English, distributed them to the research team for feedback and suggestions and then translated them into Somali for the focus groups. A focus group discussion for each study arm (cash, cash + SBCC, cash + top-up) and in both locations (Bay and Hiran) was conducted with mothers of CU5, resulting in six FGDs with mothers. One focus group in each location (Bay and Hiran), combining all three study arms, was conducted with the husbands, resulting in two men’s FGDs. Each focus group was comprised of eight participants, resulting in a total of 48 mothers and 14 husbands participating in the focus groups. The study team used the midline data set as the sampling frame for the FGDs. Key criteria used to select a representative mix of participants were maternal education status, age of the mother, and whether the mother was the head of the household. For feasibility considerations, the team also considered the distance of the participant’s household from the FGD site.

Once enrolment criteria were confirmed and informed consent was obtained, trained enumerators conducted focus group discussions with mothers and husbands of CU5 in a central location in their community. Focus groups were conducted in January 2024, about one month after the endline quantitative data collection in December 2023. The goal of these discussions was to gain additional insight and perspective into the quantitative results and better understand participants’ experiences and perceptions as well as key barriers and facilitators surrounding cash for nutrition and prevention of wasting in the study area. The mother FGDs covered the following topics: the SC Cash Plus for Nutrition program; the use of cash; health-seeking behaviours during pregnancy; child sickness and health-seeking behaviours when sick; and dietary diversity and food security. The same general topics were explored in the fathers’ FGDs, however, some of the questions had to be adapted to capture the husband/father’s perspective, such as exploring the topic of ‘supporting healthy pregnancy’ instead of ‘health-seeking behaviours during pregnancy’.

#### Qualitative data analysis

All focus group discussions were recorded and transcribed. Focus group discussion transcripts in Somali were translated into English for analysis.

An iterative mix of inductive and deductive approaches was used for analysis of the focus group discussions. Since the qualitative data collection procedure followed a structured approach, the focus group discussion guides were used to inform preliminary themes and to develop initial codes. A two-phased approach was then used to develop codes with the first few available focus group discussion transcripts. The first phase included a complete readthrough of the transcript with rapid in vivo coding, highlighting quotes and writing down any preliminary codes. Another round of coding was then done with a higher conceptual understanding, creating additional higher-level conceptual codes. Two team members engaged in this initial coding process. A codebook detailing each code (including a brief definition, a full definition, instances of when to use and when not to use each code, and an example of text that would be coded using each code) was then developed and distributed to the qualitative data analysis team [45]. An iterative, five-phased analytic cycle approach was used for analysis, consisting of the following stages: compiling, disassembling, reassembling, interpreting, and concluding [46]. At least two research team members coded each focus group discussion transcript, and coded versions of each transcript were merged to create a final coded transcript for each focus group. Key informant interviews (KII) were analysed thematically. Findings from the mothers’ focus groups, men’s focus groups, and key informant interviews were triangulated. Both exploratory analysis and explanatory sequential analysis approaches were undertaken, organising qualitative findings according to the UNICEF conceptual framework on maternal and child nutrition and triangulating the qualitative data with the quantitative results to contextualise and further explore main study findings. After analysis of qualitative data was complete, a workshop was held with the qualitative data analysis team with members from both SC Somalia and JHU to discuss, contextualise, and interpret findings. ATLAS.ti 24 software, version 25 (Scientific Software Development GmbH, Berlin, Germany) was used for analysis of the focus group discussion transcripts. Additionally, Microsoft Office Suite was used for summarising results and for KII analysis. Further details on the qualitative data methods and collection can be found in Text S15 in the **Online Supplementary Document**.

### Cost-efficiency and cost-effectiveness approach

The cost-efficiency and cost-effectiveness analyses were designed using the standards set by the Dioptra Systematic Cost Analysis Consortium. Analysis was conducted using a three-pronged, retrospective approach to data collection, including a desk review, cost estimation, and qualitative data collection from group and in-person consultations with the study implementation team. For these analyses, data was gathered regarding the program costs (financial procurement and implementation costs), project reports, results, financial reports, and survey data from the midline and endline time points. Cost inputs were estimated and linked to outputs and study outcomes in an iterative modelling process. Further discussions with in-country program staff were used to confirm estimates and assess intervention resource use, including all the direct, indirect, and ‘societal’ costs of the intervention.

Quantitative data was analysed through a combination of activity-based costing and step-down cost accounting, where the evaluator assesses transactions and assigns costs to chosen cost categories for analysis (micro-costing). Three indicators were used to evaluate the cost efficiency of each study arm: Cost per Household and Cash Transfer Ratio. The cost-effectiveness analysis compared reduction in wasting prevalences and intervention costs of Arms 2 and 3 against Arm 1 (control group). Finally, the societal cost analysis was evaluated as the ‘opportunity cost’ or time investment to beneficiary households for participating in the intervention, contributing to a holistic understanding of the intervention’s true value.

### Market monitoring

To assess the prices and availability of foods in markets throughout the study period, market monitoring activities were undertaken at markets in both Bay and Hiran (Table S10 in the **Online Supplementary Document**). Enumerators underwent a one-day training and then administered questionnaires to vendors in Bay and Hiran in July (48 vendors in Bay and 112 vendors in Hiran) and December 2023 (33 vendors in Bay and 78 vendors in Hiran). Additionally, the December questionnaire included recall questions for September 2023, in order to have market monitoring data corresponding with the baseline, midline, and endline data collection for the main quantitative component of the research study. Vendors were asked about the availability and prices of different commonly consumed foods and foods for an energy-based diet as well as reasons for fluctuations in price and availability. Enumerators supplemented the vendor questionnaires (Table S12 in the **Online Supplementary Document**) with direct observations of the markets, including taking photos, and conversations with relevant stakeholders.

Market monitoring data was analysed in Microsoft Excel and STATA. Specific food items were categorised into one of the following domains: sentinel foods, fruits and vegetables, cereals, and protein sources (Table S11 in the **Online Supplementary Document**). Trends in changes in food prices over time in both regions were analysed, and vendors’ views on the reasons for fluctuations were reported.

### Data triangulation

**Figure 3:**
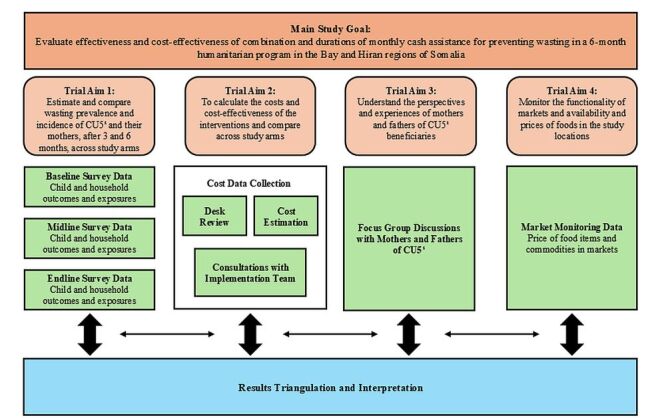
The mixed-methods approach to data collection, results triangulation and interpretation. CU5 – children under five years old.

The mixed-methods trial was designed in alignment with the trial objective and aims. Collected data was triangulated to form a holistic understanding of the study results, trial context, and participant experiences, and to inform the trial results interpretation. A figure describing the mixed-methods approach and data triangulation can be found in **Figure 3**.

### Adaptive design

The approach above describes the final adaptive design used for the trial. These methods evolved throughout the design and implementation phases of the trial as new challenges and circumstances arose. A table of study adaptations may be found in **Table 3**.

**Table 3:** Study adaptations

Study adaptation	Detailed description	Lessons learned
**Pre-specified design changes**		
Shifted research design from nine months to six months	The research team raised grant funding for nine months of the research study, however, only six months of funding from the Bureau for Humanitarian Assistance (BHA) programme were able to be used for the study. The study timeline and approach were adapted due to these funding constraints.	Close communication between the research team, implementers, donors, and governmental officials ensured that this adaptation did not delay study implementation and that changes to the research were acceptable to all involved stakeholders. Flexibility from the donor organisation also facilitated this swift timeline adaptation.
Reduced number of sampled clusters from 60 to 33	During the design stage, the research team reduced the number of sampled clusters from 60 to 44 since a limited number of clusters in the study regions met eligibility criteria for enrolment. Before baseline, an additional 11 clusters were excluded due to flooding in the Beletweyne district in Hiran. The program implementation team thought these clusters would be difficult to reach and lost to follow up. The final sample enrolled was 33 clusters (11 clusters per study arm). The total number of households enrolled was still sufficient to measure the established change in wasting prevalence.	Establishing selection criteria based on cluster vulnerability (due to climate, conflict, or other related shocks) reduced participant attrition during study implementation. This maintained the integrity of data collected and reduced participant loss to follow up.
**Iterative data-driven response**		
Adaptations to study instruments	Between midline and endline quantitative data collection, the research team added questions to the study instruments to assess seasonal flooding, displacement, and the impact this had on study participants.	Iterative adaptations to quantitative instruments based on shocks relevant to participants allowed our team to understand major concerns with trial implementation relevant to participant attrition, study outcomes, and differential impacts across trial arms. This also aided in the interpretation of study results.
**Context-driven/feasibility response**		
Data collection during seasonal flooding	Between midline and endline data collection, seasonal flooding displaced the majority of beneficiaries. In response, the country office quickly developed a follow-up team to call beneficiaries and identify their location. The number of data collectors and vehicles was increased to maximise participation at endline.	Flexibility in resource allocation allowed the study team to respond in crisis circumstances to maintain study rigour and reduce participant attrition. Contacting displaced participants by phone ensuring that enumerators were appropriately distributed to maximise participant outreach.
Modifications to the qualitative approach	The focus group discussions were originally proposed to be conducted in October 2023 during the 6-mo trial, due to flash flooding in the study area, focus groups were postponed and conducted in January 2024, about one month after endline quantitative data collection.	When working in humanitarian settings that face climate crises, it is important to be able to adapt and respond to crises in a timely way, understanding how research plans may be affected and being willing to adapt and respond while maintaining research integrity. As the qualitative data collection was an important component of the mixed-methods research, instead of dropping the focus groups, the research team instead adapted plans and timing to ensure that this data could still be collected.
Market monitoring data collection	The market monitoring data collection was adapted due to physical lack of access to markets during flooding (September 2023). At endline, vendors were asked recall questions about market prices and availability of goods during the previous months.	Finding alternative methods such as recall data supported complete data collection and a holistic understanding of market fluctuations and their impact on cash recipients.

CI – confidence interval, SD – standard deviation

### Stakeholder engagement and research uptake strategy

This methodology was co-created throughout the design and implementation of the trial through the continuous engagement of key stakeholders, which is a key feature of adaptive research methodologies [30]. This process engaged members of the research team, program staff at Save the Children, and leaders at the Ministry of Health in Somalia to ensure adaptations were acceptable and meaningful to all stakeholders involved. The methodology was co-created in an iterative process that maintained the necessary rigor and integrity of the trial while ensuring that the design would address government priorities and be practical for future researchers.

A key priority for the research team is to translate evidence into action, and the team implemented a research uptake strategy throughout the study and beyond (Text S13 in the **Online Supplementary Document**).

## DISCUSSION

This trial is one of the first to apply an adaptive mixed methods design to evaluate the effectiveness and cost-effectiveness of cash interventions in the Somalia context. The adaptive design allowed us to overcome several well-documented challenges associated with conducting research in humanitarian settings, including operational constraints, partnership coordination, participant mistrust, and instability in the study environment. In this discussion, we reflect on the key factors that enabled our study to maintain rigor while addressing these challenges and offer insights for future research in similar contexts.

One of the most critical components of the trial’s success was the strong partnership coordination between the research team, programme implementers, and local stakeholders. This close collaboration ensured that research methods were closely aligned with the realities of program implementation, allowing for necessary adaptations without compromising rigor. For example, when the trial faced a funding disparity – where research funding covered nine months but cash programming was only funded for six months – close coordination with local staff, government officials, and donors allowed us to adjust the trial timeline and maintain our research objectives within a shortened cash program. This flexibility highlights the importance of early and continuous collaboration with on-the-ground partners in humanitarian research. Such partnerships ensure that the research remains feasible and relevant to the local context while addressing logistical and operational constraints as they arise.

The adaptive nature of the trial design was another key strength. Humanitarian settings are inherently fluid, often impacted by unpredictable events such as displacement, natural disasters, or security issues. In our study, flooding and mass displacement between midline and endline data collection posed a significant challenge, threatening both the collection of endline data and the completion of focus groups. Rather than halting the study, the adaptive design enabled the team to respond quickly. We hired local village guides to locate and mobilise displaced participants, adjusted our data collection schedule, and postponed focus groups until conditions improved. These adaptations were critical to preserving the study’s integrity and minimising participant attrition, demonstrating that adaptive trial designs are essential for maintaining research rigor in unstable environments. This flexibility also required continuous communication with stakeholders, enabling the study to swiftly adjust its approach in response to emerging challenges.

Despite these successes, the complex environment of humanitarian research still presented several ongoing challenges. One notable issue was the difficulty in tracking participants for the societal cost analysis, particularly those who were displaced. Future studies should incorporate more robust tracking mechanisms and data collection strategies that account for population movement, especially in regions where displacement is common.

The success of adaptive designs in humanitarian settings is often contingent upon flexible funding models and open communication among stakeholders. In our trial, ongoing coordination with donors, implementers, and research partners, what we refer to as the ‘adaptive trifecta,’ enabled us to make real-time adjustments to the programme timeline and delivery structure in response to contextual challenges such as flooding and displacement. This level of alignment and trust was critical to preserving the study’s continuity and relevance. However, we recognise that such flexibility may not always be feasible, particularly in highly regulated or siloed funding environments. We also acknowledge the inherent tension between maintaining scientific rigor and responding to dynamic field conditions. Future research should advocate for funding structures that allow for adaptive designs and contingencies, recognising that the unpredictable nature of humanitarian settings requires a departure from traditional, rigid trial frameworks.

We summarised our main lessons learned in **Box 1**.

Box 1 **Lessons learned**

1. Early and sustained collaboration with local partners is essential to ensure that research remains feasible and contextually relevant.

2. Adaptive trial designs should be considered a valuable tool for navigating the dynamic nature of humanitarian contexts.

3. Flexibility must extend beyond the research team to include donors and funding agencies. Future research initiatives should advocate for funding models that allow for adaptive timelines and emergency contingencies to ensure studies can continue, even under evolving conditions.

## CONCLUSIONS

This study presents the mixed-methods approach used in a trial evaluating the effectiveness and cost-effectiveness of cash assistance interventions for reducing child and maternal wasting in two regions of Somalia. The adaptive methodology, flexibility and responsiveness, and strong partnerships between all relevant stakeholders were critical to the trial's success. These elements ensured that the study could navigate the complex challenges of the humanitarian context, including operational constraints and population displacement, while maintaining methodological rigor. By incorporating flexibility in both research design and funding models, future studies will be better equipped to handle unforeseen challenges while producing reliable and actionable findings. In conclusion, the methodology and experiences shared here will contribute to more responsive and resilient research in humanitarian settings, ensuring that future studies are both rigorous and aligned with the needs of those most affected by crises.

## Additional material


Online Supplementary Document
